# Comparison of cardiovascular disease risk association with metabolic unhealthy obesity identified by body fat percentage and body mass index: Results from the 1999–2020 National Health and Nutrition Examination Survey

**DOI:** 10.1371/journal.pone.0305592

**Published:** 2024-08-14

**Authors:** Qian Xiong, Yang Zhang, Jun Li, Yaping An, Shan Yu

**Affiliations:** Department of Cardiology, Guizhou Provincial People’s Hospital, Guiyang, China; Endocrinology and Metabolism Population Sciences Institute, Tehran University of Medical Sciences, ISLAMIC REPUBLIC OF IRAN

## Abstract

**Background and aim:**

Cardiovascular disease (CVD) risk among individuals across different categories of metabolic obesity phenotypes is controversial. The study used body fat percentage (BFP) or body mass index (BMI) to categorize obese status and to investigate the association between metabolic obesity phenotypes and CVD risk in a nationally representative population.

**Methods:**

This cross-sectional study included 49463 adult participants in National Health and Nutrition Examination Survey from 1999 to 2020. Metabolic healthy status was defined by the absence of metabolic syndrome according to the revised National Cholesterol Education Program Adult Treatment Group definition. Obesity was identified by BFP, assessed by dual-energy X-ray absorptiometry scan, and BMI. The primary outcome was CVD prevalence. The multivariable logistic regression model and restricted cubic spline analyses were used to examine the associations between metabolic obesity phenotypes and the risk of CVD.

**Results:**

Among 49463 adult participants, 32.12% were metabolically unhealthy, 34.10% were overweight, 37.94% were obese; and 8.41% had CVD. Compared with metabolic healthy normal weight, metabolic healthy obesity, and metabolic unhealthy normal weight/overweight/obesity were all associated with increased CVD risk with adjusted odds ratios (95% confidence intervals) of 1.45 (1.14–1.85), 2.80(1.53–5.11), 2.55(1.88–3.47), and 2.96(2.18–4.02), respectively. Nonlinear dose-response relationships between BFP and CVD were observed both in metabolically healthy and unhealthy participants (both *P* for non-linearity<0.0001). When obesity was defined with BMI, there were a similar prevalence of obesity, and similar associations between metabolic obesity phenotypes and CKD risks.

**Conclusions:**

Metabolic healthy and unhealthy obesity were both associated with higher risks of CVD, whether using BFP or BMI to define obese status. It suggests that metabolic obesity phenotype is a risk factor for CVD.

## Introduction

As the global population ages and new epidemiological changes arise, cardiovascular disease (CVD) remains the leading cause of death worldwide, threatening human health [1, 2]. The number of people with CVD has nearly doubled in the past 30 years, from 271 million in 1990 to 523 million in 2019. This increase has resulted in a staggering 18.6 million deaths in 2019 [2]. Obesity is a worldwide epidemic, with over 1.9 billion overweight adults, and 650 million are obese, accounting for almost 40% of adults worldwide [3]. Obesity is considered a critical independent risk factor for CVD, worsening most risk factors, including adverse effects on blood pressure, blood, lipid metabolism, and inflammation, further impairing cardiac structure and function [4, 5]. It is important to note that two-thirds of deaths related to obesity are caused by CVD [6].

Obesity is often accompanied by metabolic syndrome (MetS) [7], which increases their risk of CVD [8]. However, not all individuals with obesity have metabolic disorders, such as MetS. Metabolic phenotypes have been categorized into metabolically healthy normal weight (MHN), metabolically healthy overweight/obesity (MHOW/MHO), metabolically unhealthy normal weight (MUN), and metabolically unhealthy overweight/obesity (MUOW/MUO), based on the concept of metabolic health and adiposity status [9]. It has been observed that some individuals with MHO do not have an increased risk of developing CVD [10–12]. However, some studies have found that MHO is associated with negative CVD outcomes [13–15]. Additionally, a few researches suggest that individuals with MUO present the highest risk of CVD when compared to other subsets [16, 17]. Distinct disease outcomes in individuals with different phenotypes may be attributed to the absence of consistent criteria to define metabolic phenotypes or discrepancies in sample size and ethnicity [10–17]. Previous studies commonly assessed obesity with body mass index (BMI), however, BMI is not a perfect indicator of obesity, considering that BMI could not effectively differentiate between muscle and fat mass [18]. This limitation could have influenced the previous results, and a more precise method is required to assess body fat. Dual-energy x-ray absorptiometry (DXA) is a method that evaluate body fat with higher accuracy than BMI [19].

Hence, to further understand the effects of metabolic health and adiposity on CVD risks, we used body fat percentage (BFP) assessed by DXA scans to categorize obese status, to examine the associations of metabolic phenotypes with risks of CVD prevalence.

## Methods

### Study population

**Fig 1:**
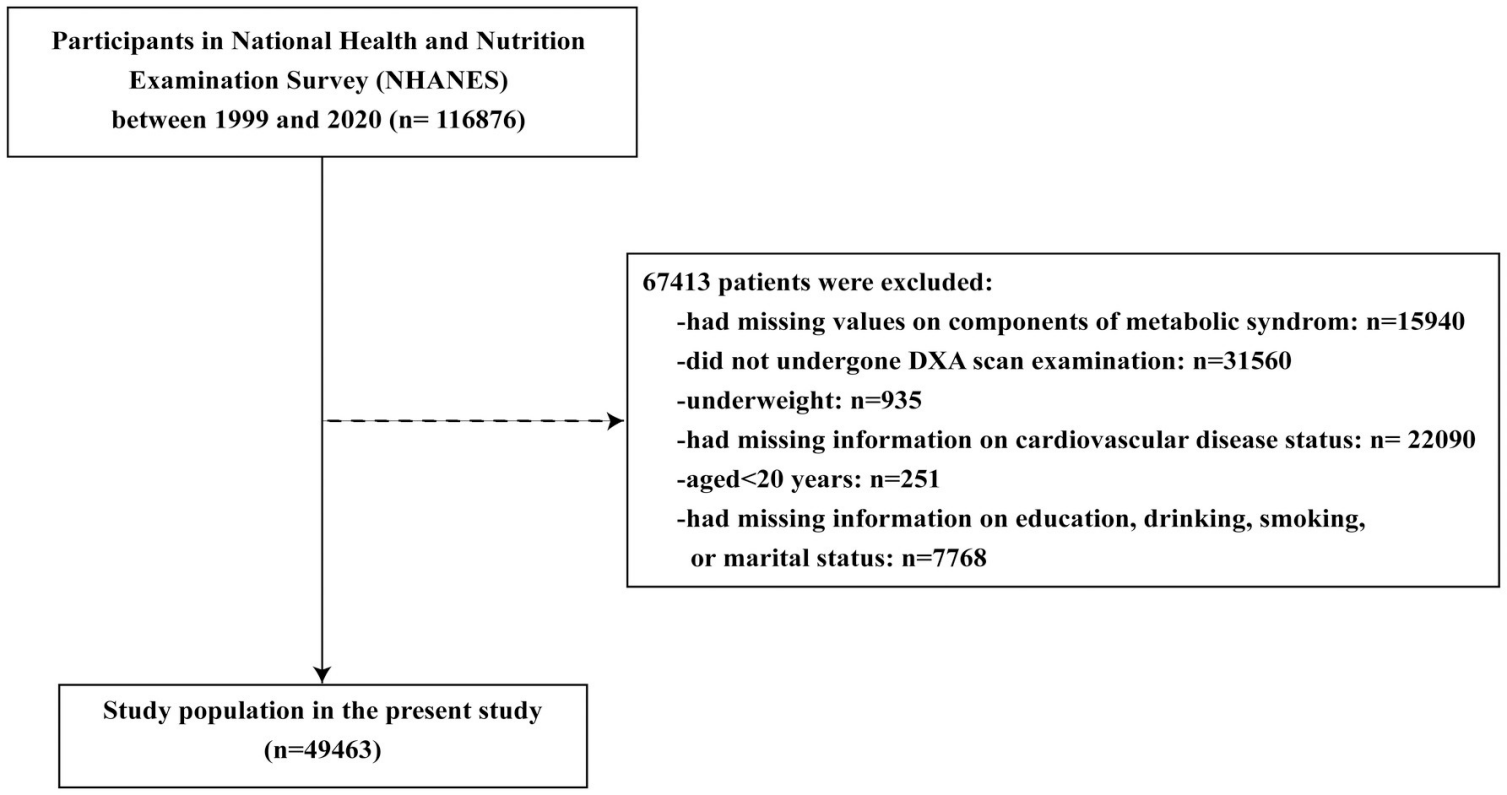
Flowchart of this study.

This study was conducted using data from eleven consecutive cycles of the National Health and Nutrition Examination Survey (NHANES) conducted by the National Centre for Health Statistics (NCHS) in the United States. The survey is ongoing and aims to evaluate the nutritional and health status of the non-institutionalized US civilian population. It is conducted every two years and comprises a structured interview conducted at home, followed by a standardized health examination that includes physical examination and laboratory tests. All of them can be publicly available at http://www.cdc.gov/nchs/nhanes/. The NHANES official website provides guidelines and regulations for data processing and statistical analysis. For this study, we included participants enrolled from 1990 to 2020 and excluded those who were under 20 years of age, had missing values for MetS components, did not undergo DXA scan examination or had missing BMI values, were underweight, had missing values for CVD status, or had missing information on age, sex, ethnic, education, marital status, smoking, or drinking status. Fig 1 shows the selection process in detail.

NHANES follows standardized protocols that have been approved by the institutional review board of the Centers for Disease Control and Prevention to collect biological samples for laboratory analyses. The NHANES surveys and examinations obtained written informed consent from all participants after receiving approval from the National Center for Health Statistics Research Ethics Review Board. The study was conducted by the Declaration of Helsinki. This cross-sectional study followed the Strengthening the Reporting of Observational Studies in Epidemiology (STROBE) reporting guideline for reporting and analyses. Additional information on the study design is available at https://www.cdc.gov/nchs/nhanes/index.htm.

### Data collection

Data on demographic characteristics, educational level, smoking and drinking status, and physical activities were collected using standardized questionnaires. Height, weight, waist circumference, and systolic, and diastolic blood pressure were measured using calibrated instruments with standard protocols by trained staff during the mobile examination center visit. A subset of participants provided fasting venous blood samples, which were used to measure total cholesterol (CHOL), low-density lipoprotein cholesterol (LDL-c), high-density lipoprotein cholesterol (HDL-c), triglyceride (TG), and fasting plasma glucose (FPG) using automated methods.

The whole-body DXA examinations were conducted at the mobile examination center (MEC) by certified radiology technologists with a Hologic QDR-4500A fan-beam densitometer (Hologic, Inc., Bedford, Massachusetts). The participants were positioned on the tabletop in a supine position with their feet in a neutral position and hands flat by their sides. The DXA technique acquires two low-dose X-ray images at different average energies. The ratio of the attenuation of these two average energies is used to distinguish both bone from soft tissue, and the percentage of fat in soft tissue when bone isn’t present. Each DXA scan was reviewed and analyzed by the Department of Radiology of University of California, San Francisco, using standard radiologic techniques and study-specific protocols developed for the NHANES. Hologic Discovery software version 12.1 was used to analyze data. Body fat percentage was determined as a ratio of fat mass over total body mass (including bone mineral mass). Further details of the DXA examination protocol are documented in the Body Composition Procedures Manual located on the NHANES website (https://wwwn.cdc.gov/nchs/data/nhanes/2005-2006/manuals/bc).

### Assessment of obesity and metabolic syndrome phenotypes

Metabolic health was defined by the absence of metabolic syndrome (MetS) according to the revised National Cholesterol Education Program Adult Treatment Group (NCEP-ATP III) definition [20]. According to the MetS definition, individuals were deemed metabolically healthy if they had no more than two of the following disorders: systolic blood pressure ≥ 130 mmHg or diastolic blood pressure ≥ 85 mmHg, or use of antihypertensive medication; TG level ≥ 1.70 mmol/L; HDL-C level < 1.03 mmol/L in men or < 1.29 mmol/L in women; and FPG level ≥ 5.60 mmol/L or previously diagnosed with diabetes; waist circumference ≥ 90 cm in men or ≥ 80 cm in women. BFP-based criteria recommended by Bray were used to classify obese status into normal weight, overweight, or obesity (S1 Table) [21].

Participants were divided into six subgroups: metabolically healthy normal weight (MHN), metabolically healthy overweight (MHOW), MHO, metabolically unhealthy normal weight (MUN), metabolically unhealthy overweight (MUOW), and metabolically unhealthy obesity (MUO).

### Assessment of cardiovascular disease

The term CVD refers to a self-reported diagnosis of five major cardiovascular diseases, which are congestive heart failure (CHF), coronary heart disease (CHD), angina pectoris, heart attack, and stroke. This diagnosis was obtained through a standardized medical status questionnaire that participants completed during individual interviews. The questionnaire asked participants if they had ever been informed by a physician about having any of the five CVD events. Those who responded positively were considered to have CVD. You can find more information about this at the following URL: [https://wwwn.cdc.gov/Nchs/Nhanes/2003-2004/MCQ_C.htm].

### Assessment of covariates

The study considered several potential factors that could affect the results. These factors included age, sex, race/ethnicity, education level, and marital status. Health behaviors such as smoking, drinking, and physical activity were also taken into account. Smoking status were divided into three group, including never smoker, former smoker and current smoker, based on the self-reported questionnaire information. Those who had not smoked 100 cigarettes in their lifetime were classified as never-smokers, and those who had smoked 100 cigarettes in their lifetime but had quitted smoking now were defined as former smokers, while those who had smoked 100 cigarettes in their lifetime and were still smoking now were defined as current smokers. Similarly, drinking status was classified into three categories, including never drinkers, former drinkers, and current drinkers. Those who had not get 12 drinks in their lifetime were defined as never-drinkers, and those who had 12 drinks in their lifetime but had quitted drinking were classified as former drinkers, and those who had 12 drinks in their lifetime and were still having at least one drink during the past 12 months were defined as current drinkers. physical activity was assessed using the physical activity questionnaire, which asked participants about the frequency and duration of vigorous and moderate physical activities, workouts, and leisure activities lasting at least ten consecutive minutes per week. Physical activity was calculated using the number of times per week and length of time. The “Active” was defined was at least 150 min per week of total physical activity in more than 2 sessions, otherwise, was defined as “Inactive”. Hypertension was defined as having an average systolic blood pressure above 140 mmHg or an average diastolic blood pressure above 90 mmHg, self-reported history of hypertension, or taking antihypertensive drugs. Diabetes was defined as having fasting blood glucose levels of 7.0 mmol/L or higher, random blood glucose levels of 11.1 mmol/L or higher, self-reported history of diabetes, or taking antidiabetic drugs.

### Statistical analyses

The data collected in NHANES were obtained through a stratified, multistage probability sample design. Therefore, we used survey analysis procedures to account for sample weights, stratification, and clustering in our study. Weights, created by the CDC, account for the complex survey design of NHANES (including oversampling), survey non-response and poststratification adjustment to match total population counts from the USA. According to the NHANES analysis guidelines, in our study, data from 1998 to 2020 were combined; we constructed combined sample weights using 4-year weight from 1999 to 2002, 2-year weight from 2023 to 2016, and the special sample weights were used for NHANES 2017–2020 pre-pandemic data. Detailed instructions for combining datasets from the NHANES cycles are provided in the NHANES Analytic Guidelines: https://wwwn.cdc.gov/nchs/nhanes/tutorials/weighting.aspx.

We described the sample size and characteristics based on different metabolic health phenotypes. The data were presented as weighted proportions (95% confidence intervals, CIs) for categorical variables and weighted means (95%CIs) for continuous variables. To compare across metabolic obesity phenotypes, continuous variables were compared using the survey-weighted linear regression, and categorical variables were compared using the survey-weighted Chi-square test (statistic = adjWald). Weighted univariate and multivariate logistic regression were used to identify independent covariates and estimate the effect of different phenotypes on the risk of CVD, with odds ratios (ORs) and 95% CIs. We used three models for the logistic regression analysis. In model 1, there was no adjustment. In model 2, we adjusted for age and sex. In model 3, we adjusted for age, sex, ethnicity, education level, marital status, physical activity, smoking, and drinking status.

To explore the dose-response relationship between BFP and CVD in metabolically healthy and unhealthy participants, restricted cubic splines (RCS) were deployed. Various knot placements between 3 and 7 were tested, with the model featuring the lowest Akaike Information Criterion value selected for RCS, ultimately utilizing 4 knots. The inflection point was determined based on the shape of the RCS.

The multivariable-adjusted model was stratified by demographic and lifestyle characteristics as potential modifiers: age (<40 or ≥40 years), sex (female or male), smoking status (never, former, or now), drinking (never, former or now), physical activity (inactive or active). The multiplicative interaction terms between these subgroups and metabolic health-obesity phenotypes were added to the fully adjusted model, and models with and without multiplicative interaction terms were compared using the likelihood ratio test.

In order to fully investigate the link between metabolic phenotypes and CVD, we used BMI criteria to define obesity status. We further classified individuals into three categories based on their BMI: normal weight (BMI 18.5- <25 kg/m^2^), overweight (BMI 25–<30 kg/m^2^), and obesity (BMI ≥ 30 kg/m^2^), in line with the World Health Organization’s recommendations [22].

All statistical analyses were performed using the statistical package R (The R Foundation; http://ww.r-project.org; version 4.2.1). The “survey” package was employed [23]. Two-tailed tests were used and the *P* value <0.05 was considered statistically different.

## Results

### Baseline characteristics of the study population

This study enrolled 49463 adult participants in the final analyses. The mean age of participants was 46.9 years, and 49.28% were male. Of all participants, 32.12% were metabolically unhealthy, and 34.10% were overweight, 37.94% were obese. Based on metabolic health and obese status, 16.96% of participants were MHN, 22.64% MHOW, 28.28% MHO, 0.44% MUN, 4.61% MUOW, and 27.06% MUO. Baseline characteristics of participants in six subgroups are shown in Table 1. Compared with those with MHN, participants with the other five subgroups tended to be older, had lower educational levels, were more likely to drink alcohol, smoke, and be inactive; were more likely to have a history of diabetes, hypertension, hyperlipidemia; and tended to have higher blood pressure levels and higher glucose levels.

**Table 1:** Baseline characteristics of all participants categorized by metabolic obesity phenotypes (defined by BFP).

Characteristics	Overall	MHN	MHOW	MHO	MUN	MUOW	MUO	*P*
No. of participants	49463	12811(25.90)	12046(24.35)	8718(17.63)	1019(2.06)	4822(9.75)	10047(20.31)	
Age, year	46.9 (46.6–47.2)	36.6 (36.3–36.9)	47.6 (47.2–48)	46.5 (46.2–46.8)	49.6 (47.8–51.4)	54.3 (53.8–54.8)	53.5 (53.1–53.9)	<0.001
Male (%)	49.2 (49.2–49.2)	43.5 (42.4–44.6)	52.1 (50.9–53.3)	52.5 (51.7–53.3)	33.6 (23.0–44.2)	36.9 (34.7–39.1)	49.2 (47.9–50.5)	<0.001
Hispanic (%)								<0.001
Mexican American	8.2 (8.2–8.2)	6.1 (5.5–6.6)	7.6 (7–8.2)	9.7 (8.8–10.6)	3.2 (2.3–4.0)	8.2 (7.1–9.2)	8.6 (7.9–9.2)	
Non-Hispanic Black	10.7 (10.7–10.7)	9.8 (9.2–10.5)	9.5 (8.9–10)	14.3 (13.3–15.3)	7.4 (5.5–9.4)	6.2 (5.5–6.8)	9.2 (8.4–9.9)	
Non-Hispanic White	67.2 (67.2–67.2)	68.1 (66.9–69.3)	67.9 (66.5–69.3)	63.6 (62.2–65.0)	72.2 (68.8–75.6)	67.9 (65.8–70.0)	70.2 (68.9–71.5)	
Other Hispanic	6.4 (6.4–6.4)	5.8 (5.3–6.3)	6.9 (6.2–7.5)	6.8 (6.3–7.4)	3.5 (2.4–4.7)	7.1 (6.2–8.0)	5.8 (5.3–6.4)	
Other races	7.4 (7.4–7.4)	10.1 (9.5–10.7)	8.1 (7.5–8.8)	5.4 (5.1–5.7)	13.6 (10.8–16.4)	10.6 (9.4–11.8)	6.2 (5.7–6.7)	
Education (%)								<0.001
Less than 11 grade	13.7 (13.7–13.7)	11.0 (10.3–11.7)	13.2 (12.7–13.7)	12.6 (12.1–13.1)	20.5 (17.4–23.6)	19.6 (18.4–20.8)	16.4 (15.8–17.0)	
High school graduate	30.2 (30.2–30.2)	36.3 (34.7–37.9)	36.3 (34.8–37.8)	30.0 (28.8–31.2)	24.4 (14.2–34.6)	22.7 (20.4–25.0)	21.2 (20.3–22.1)	
Some college	24.9 (24.9–24.9)	22 (21.2–22.8)	21.8 (20.8–22.8)	24.5 (23.6–25.4)	35.1 (24.5–45.7)	30.6 (28.6–32.6)	29.4 (28.7–30.1)	
College graduate or above	31.1 (31.1–31.1)	30.5 (29.5–31.5)	28.4 (27.6–29.2)	32.7 (32.1–33.4)	19.8 (16.7–22.9)	26.9 (25.2–28.6)	32.9 (32.2–33.6)	
Married (%)	59.7 (59.7–59.7)	48.3 (47.3–49.3)	63.4 (62.5–64.3)	60.2 (59.3–61.1)	47.6 (37.1–58.1)	63.9 (62.1–65.7)	64.0 (63.0–65.0)	<0.001
Smoking status (%)								<0.001
Never	55.3 (55.3–55.3)	57.0 (55.9–58.1)	55.4 (54.3–56.5)	58.6 (57.4–59.8)	39.6 (29.0–50.2)	47.9 (46.1–49.7)	51.2 (50.5–51.9)	
Former	25.1 (25.1–25.1)	16.3 (15.2–17.4)	24.8 (23.8–25.8)	25.3 (24.4–26.2)	15.9 (13.4–18.4)	24.1 (22.3–25.9)	32.2 (31.2–33.2)	
Current	19.6 (19.6–19.6)	26.5 (25.4–27.6)	19.7 (18.6–20.8)	16.0 (15.3–16.7)	44.4 (33.9–54.9)	27.9 (26.0–29.8)	16.5 (15.8–17.2)	
Drinking status (%)								<0.001
Never	10.0 (10.0–10.0)	9.8 (9.1–10.6)	9.0 (8.5–9.5)	8.9 (8.5–9.4)	10.3 (8.1–12.5)	14.4 (13.1–15.7)	11.8 (11.2–12.4)	
Former	8.6 (8.6–8.6)	4.8 (4.5–5.2)	7.6 (7.3–8.0)	7.6 (7.3–8.0)	14.1 (11.9–16.3)	13.5 (12.4–14.6)	12.6 (12–13.2)	
Current	81.3 (81.3–81.3)	85.3 (84.4–86.2)	83.3 (82.6–84.0)	83.4 (82.8–84.0)	75.5 (72.5–78.5)	72.0 (70.3–73.7)	75.5 (74.7–76.3)	
Active (%)	48.4 (48.4–48.4)	50.9 (49.9–51.9)	48.9 (48–49.8)	49.6 (48.5–50.7)	51.5 (48.2–54.8)	40.6 (38.9–42.3)	45.7 (44.8–46.6)	<0.001
Central obesity (%)	74.5 (74.5–74.5)	11.1 (10.5–11.7)	72.6 (71.6–73.6)	98.7 (98.6–98.9)	63.5 (60.3–66.7)	94.4 (93.6–95.2)	99.9 (99.9–99.9)	<0.001
SBP, mmHg	72.7 (72.6–72.8)	69.1 (68.8–69.3)	71.8 (71.6–72)	73.8 (73.6–74)	77.8 (75.1–80.5)	72.8 (72.4–73.2)	74.8 (74.6–75)	<0.001
DBP, mmHg	121 (120.8–121.2)	114 (113.7–114.3)	121 (120.7–121.3)	122.0 (121.7–122.3)	130.0 (128.5–131.5)	125.0 (124.3–125.7)	127.0 (126.7–127.3)	<0.001
BMI, kg/m^2^	29.3 (29.2–29.4)	21.8 (21.8–21.8)	25.5 (25.5–25.5)	33.1 (33–33.2)	22.2 (22.1–22.3)	25.8 (25.7–25.9)	34.9 (34.8–35.0)	<0.001
WC, cm	99.4 (99.2–99.6)	79.9 (79.7–80.1)	90.8 (90.7–90.9)	107.0 (106.8–107.2)	87.7 (87.0–88.4)	95.3 (95.1–95.5)	114.0 (113.7–114.3)	<0.001
Diabetes (%)	19.6 (19.6–19.6)	3.7 (3.3–4.1)	9.9 (9.3–10.4)	10.9 (10.2–11.6)	43.1 (32.6–53.6)	38.4 (36.4–40.4)	48.8 (47.9–49.7)	<0.001
Hypertension (%)	34.3 (34.3–34.3)	12 (11.4–12.6)	24.2 (23.5–24.9)	29.5 (28.7–30.3)	68.2 (64.5–71.9)	56.6 (54.8–58.4)	63.3 (62.3–64.3)	<0.001
Hyperlipidemia (%)	67.4 (67.4–67.4)	40.4 (39.2–41.6)	63.2 (62.1–64.3)	62.7 (61.9–63.5)	95.6 (93.9–97.3)	96.0 (95.1–96.9)	93.4 (93–93.8)	<0.001
CHOL, mmol/L	5.0 (5.0–5.0)	4.7 (4.7–4.8)	5.1 (5.1–5.1)	5 (5.0–5.1)	5.3 (5.1–5.6)	5.3 (5.2–5.3)	5.0 (5.0–5.1)	<0.001
TG, mmol/L	1.6 (1.6–1.7)	1.1 (1.1–1.1)	1.4 (1.3–1.4)	1.4 (1.4–1.4)	2.8 (2.5–3.0)	2.7 (2.6–2.8)	2.5 (2.4–2.5)	<0.001
HDL-C, mmol/L	1.4 (1.4–1.4)	1.6 (1.6–1.6)	1.5 (1.5–1.5)	1.4 (1.4–1.4)	1.2 (1.2–1.3.)	1.2 (1.1–1.2)	1.1 (1.1–1.1)	<0.001
LDL-C, mmol/L	3.0 (2.9–3.0)	2.7 (2.7–2.7)	3.0 (3.0–3.1)	3.1 (3.0–3.1)	2.9 (2.6–3.1)	3.1 (3.1–3.2)	2.9 (2.9–3.0)	<0.001
FBG, mmol/L	5.4 (5.4–5.5)	4.9 (4.9–4.9)	5.1 (5.1–5.2)	5.2 (5.2–5.2)	7.7 (6.3–9.0)	6.0 (5.9–6.1)	6.3 (6.3–6.3)	<0.001

*P*<0.05 was considered statistically significant. Values were expressed as mean, or prevalence (95%CI). MHN, metabolically healthy normal weight; MHOW, metabolically healthy overweight; MHO, metabolically healthy obesity; MUN, metabolically unhealthy normal weight; MUOW, metabolically unhealthy overweight; MUO, metabolically unhealthy obesity; SBP, systolic blood pressure; DBP, diastolic blood pressure; BMI, body mass index; WC, waist circumference; CHOL, total cholesterol; TG, triglycerides; HDL-C, high density lipoprotein cholesterol; LDL-C, low density lipoprotein cholesterol; FBG, fasting blood glucose.

Among all participants, 8.41% had CVD, including 2.77% stroke, 2.21% CHF, 2.33% angina, 3.27% heart attack, and 3.51% CHD. For CVD, the weighted prevalence in MHN, MHOW, and MHO groups were 2.21%, 6.18%, and 6.61%; while the risks were relatively higher in metabolically unhealthy subgroups, with the weighted prevalence increased to 12.18%, 14.81%, and 16.53% in MUN, MUOW, and MUO subgroups (Table 2). For stroke, CHD, CHF, angina, and heart attack, the outcome differences in prevalence were similar, with the MUO group having the highest risk of CVD events, while the MHN group had the lowest risk (Table 2).

**Table 2:** Prevalence of CVD of all participants categorized by metabolic obesity phenotypes.

Characteristics	Overall	MHN	MHOW	MHO	MUN	MUOW	MUO	*P*-value
CVD	8.4 (8.4–8.4)	2.2 (2.0–2.4)	6.2 (5.8–6.6)	6.6 (6.3–6.9)	12.1 (9.5–14.7)	14.8 (13.5–16.1)	16.5 (15.6–17.4)	<0.001
Stroke	2.8 (2.8–2.8)	0.9 (0.8–1.1)	1.7 (1.5–1.9)	2.6 (2.4–2.8)	4.5 (2.9–6.1)	4.0 (3.5–4.5)	5.2 (4.8–5.7)	<0.001
Angina	2.3 (2.3–2.3)	0.5 (0.4–0.6)	1.7 (1.4–2.0)	1.6 (1.5–1.8)	2.0 (1.4–2.5)	4.2 (3.1–5.3)	4.9 (4.4–5.4)	<0.001
CHD	3.5 (3.5–3.5)	0.5 (0.4–0.6)	2.7 (2.4–3.0)	2.1 (1.9–2.3)	4.6 (2.9–6.4)	7.7 (6.6–8.8)	7.7 (7.0–8.5)	<0.001
Heart attack	3.3 (3.3–3.3)	0.8 (0.6–1.0)	2.2 (2.0–2.5)	2.4 (2.2–2.6)	4.1 (2.7–5.4)	5.4 (4.6–6.2)	7.0 (6.4–7.5)	<0.001
CHF	2.2 (2.2–2.2)	0.4 (0.3–0.5)	1.2 (1.0–1.4)	1.5 (1.4–1.6)	2.2 (1.4–3.1)	3.5 (2.9–4.0)	5.2 (4.8–5.7)	<0.001

P<0.05 was considered statistically significant. Values were expressed as mean, or prevalence (95%CI). MHN, metabolically healthy normal weight; MHOW, metabolically healthy overweight; MHO, metabolically healthy obesity; MUN, metabolically unhealthy normal weight; MUOW, metabolically unhealthy overweight; MUO, metabolically unhealthy obesity; CVD, cardiovascular disease; CHD, coronary heart disease; CHF, congestive heart failure.

### Association of metabolic phenotypes with CVD prevalence

Table 3 presents the association between metabolic phenotypes, defined by BFP, and CVD prevalence. After adjusting for confounding factors, the risk of CVD was found to be 45% higher in the MUO group (OR: 1.45, 95% CI: 1.14–1.85) compared to the MHN group. However, the MHOW group did not show a significantly higher risk of CVD (OR: 1.29, 95% CI: 0.97–1.73). For metabolically unhealthy individuals, all three subgroups were associated with a higher risk of CVD with corresponding ORs (95% CIs) of 2.80 (1.53–5.11), 2.55 (1.88–3.47), and 2.96 (2.18–4.02), respectively. Similar results were observed when five single CVD events were the outcome events (Table 3).

**Table 3:** Associations between metabolic obesity phenotypes and CVD risk.

Variables	MHN	MHOW	MHO	MUN	MUOW	MUO
**CVD**						
Model 1	Reference	2.92(2.23, 3.82)	3.14(2.52, 3.91)	6.15(3.59,10.53)	7.70(5.83,10.17)	8.78(6.55,11.77)
Model 2	Reference	1.19(0.90,1.56)	1.32(1.04,1.66)	3.18(1.64,6.14)	2.58(1.93,3.44)	2.86(2.12,3.85)
Model 3	Reference	1.29(0.97,1.73)	1.45(1.14,1.85)	2.80(1.53,5.11)	2.55(1.88,3.47)	2.96(2.18,4.02)
**Stroke**						
Model 1	Reference	1.83(1.24, 2.69)	2.83(2.05, 3.90)	4.96(2.19,11.21)	4.38(2.80, 6.84)	5.80(4.06, 8.27)
Model 2	Reference	0.85(0.59,1.22)	1.36(1.00,1.85)	2.53(1.07,6.00)	1.52(0.99,2.33)	2.07(1.45,2.95)
Model 3	Reference	0.91(0.61,1.34)	1.48(1.05,2.08)	2.02(0.86,4.77)	1.42(0.91,2.22)	2.13(1.45,3.12)
**CHD**						
Model 1	Reference	5.48(3.14, 9.58)	4.20(2.52, 6.98)	9.54(3.83,23.78)	16.35(9.14,29.27)	16.41(9.37,28.73)
Model 2	Reference	1.90(1.07, 3.38)	1.45(0.87, 2.41)	4.99(1.79,13.91)	5.02(2.75, 9.19)	4.42(2.46, 7.92)
Model 3	Reference	1.99(1.12, 3.54)	1.51(0.90, 2.52)	4.92(1.81,13.39)	5.11(2.77, 9.44)	4.40(2.48, 7.81)
**CHF**						
Model 1	Reference	2.83(1.74, 4.61)	3.51(2.31, 5.35)	5.27(2.32,11.96)	8.35(5.24,13.31)	12.82(8.52,19.27)
Model 2	Reference	1.13(0.68,1.87)	1.43(0.92,2.22)	2.69(1.13,6.41)	2.69(1.62,4.48)	3.94(2.52,6.14)
Model 3	Reference	1.21(0.74,1.98)	1.52(0.98,2.35)	2.28(0.97,5.34)	2.56(1.55,4.22)	3.85(2.51,5.90)
**Heart attack**						
Model 1	Reference	2.86(1.70, 4.82)	3.07(2.07, 4.55)	5.33(2.36,11.99)	7.14(4.43,11.49)	9.37(6.18,14.19)
Model 2	Reference	1.18(0.68,2.04)	1.29(0.84,1.98)	2.89(1.18,7.08)	2.54(1.53,4.21)	3.04(1.93,4.78)
Model 3	Reference	1.34(0.77,2.33)	1.53(1.01,2.32)	2.56(1.09,6.04)	2.55(1.56,4.18)	3.29(2.11,5.12)
**Angina**						
Model 1	Reference	3.27(1.89, 5.66)	3.15(1.99, 4.96)	3.79(1.96, 7.34)	8.25(4.07,16.70)	9.76(6.07,15.71)
Model 2	Reference	1.47(0.85,2.54)	1.44(0.93,2.22)	2.04(1.00,4.16)	3.05(1.48,6.30)	3.41(2.19,5.30)
Model 3	Reference	1.60(0.93,2.77)	1.62(1.05,2.50)	1.84(0.91,3.72)	3.00(1.40,6.42)	3.51(2.24,5.50)

MHN, metabolically healthy normal weight; MHOW, metabolically healthy overweight; MHO, metabolically healthy obesity; MUN, metabolically unhealthy normal weight; MUOW, metabolically unhealthy overweight; MUO, metabolically unhealthy obesity; CVD, cardiovascular disease; CHD, coronary heart disease; CHF, congestive heart failure.

Model 1, unadjusted; Model 2, adjusted for age, and sex; Model 3, adjusted for age, sex, ethnicity, educational level, marital status, smoking status, drinking status, and physical activity.

**Fig 2:**
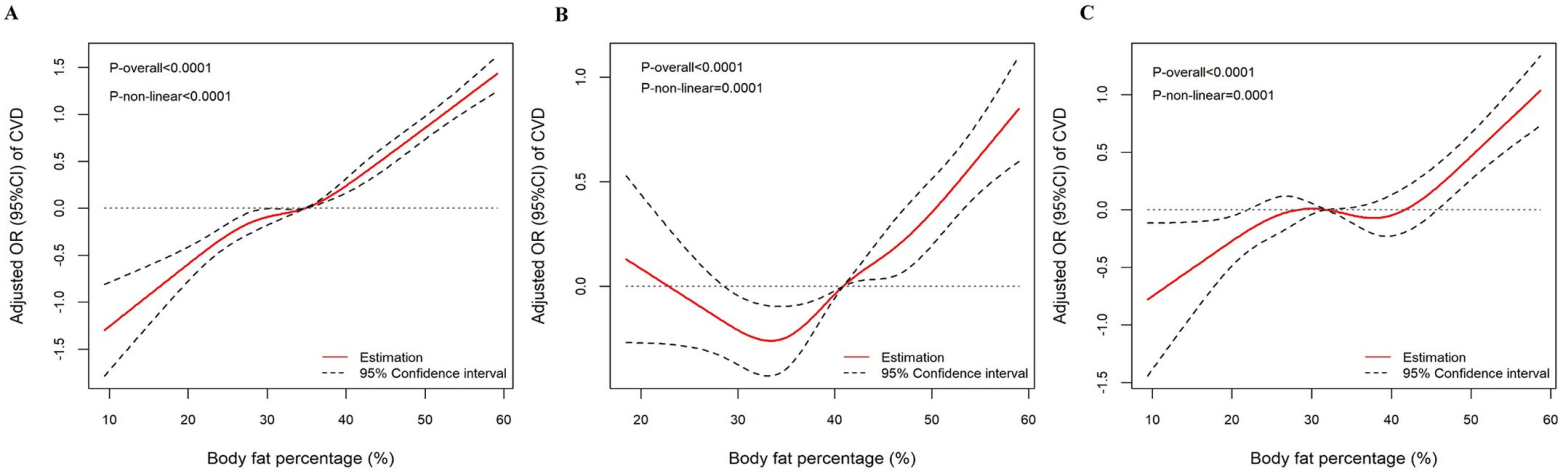
Dose-response relationship between BFP level and CVD. The association between BFP level and CVD among all participants (A), metabolically healthy (B), and unhealthy (C) participants. The solid lines and shaded areas represent the ORs and corresponding 95% CIs. The models were adjusted for age, sex, ethnicity, educational level, marital status, smoking status, drinking status, and physical activity. P-values for non-linearity were obtained using a chi-squared test to compare nested models. CVD, cardiovascular disease; OR, odds ratio; CI, confidence interval.

Fig 2 depicts the results of restricted spline analyses. Non-linear dose-response relationships between BFP and CVD were observed in all participants, whether metabolically healthy or unhealthy. These relationships were statistically significant with (all P for non-linearity < 0.001). For all participants, the threshold was 36.8%, BFP above the threshold was associated with higher risk of CVD (Fig 2A). For participants with metabolically healthy, the threshold range of BFP was 22.4%-40.8%. Lower and higher BFP levels were both associated with higher risk of CVD (Fig 2B). For participants with metabolically unhealthy, the threshold range of BFP was 30.6%-42.2%. Higher BFP levels were both associated with higher risk of CVD, whether below or above the threshold range (Fig 2C).

Based on BMI criteria, the sensitivity analyses revealed that 25.90% participants were categorized as MHN, 24.35% as MHOW, 17.63% as MHO, 2.06% as MUN, 9.75% as MUOW, and 20.31% as MUO. The associations between the different phenotypes and CVD remained largely unchanged when using BMI categories as the diagnosis criteria for obese status, as shown in S2 Table. Additionally, there was no interaction between metabolic phenotypes and sex with respect to the risk of CVD, as shown in S3 Table.

### Subgroup analyses

Subgroup analyses are presented in Table 4. The associations of metabolic phenotypes with CVD prevalence were more robust among individuals who were younger than 40, female, White, smoker, drinker, and inactive (all *P*<0.05). The interaction of metabolic phenotypes with sex on the risk of CVD was detected (all *P* for interaction = 0.04), while no interactions with age, physical activity, smoking status, and drinking status (all *P* for interaction >0.05).

**Table 4:** Subgroup analyses of association between metabolic obesity phenotypes and CVD risk.

Variables	MHN	MHOW	MHO	MUN	MUOW	MUO	*P* for interaction
**Age**							0.23
≥40 years	Reference	1.92(1.42,2.58)	2.31(1.81,2.94)	2.93(1.53,5.59)	3.84(2.86,5.16)	4.46(3.21,6.21)	
<40 years	Reference	1.34(0.79, 2.26)	1.42(0.82, 2.46)	5.91(1.25,27.84)	1.08(0.22, 5.39)	4.75(2.39, 9.45)	
**Sex**							0.04
Female	Reference	1.63(1.11,2.38)	2.35(1.61,3.42)	4.36(2.23,8.53)	3.05(1.98,4.71)	4.73(2.91,7.69)	
Male	Reference	1.88(1.30,2.72)	1.96(1.41,2.72)	1.39(0.50,3.84)	4.22(2.62,6.78)	3.83(2.80,5.22)	
**Ethnicity**							0.08
Mexican American	Reference	0.87(0.35,2.15)	1.13(0.50,2.57)	0.89(0.19,4.32)	2.49(1.03,5.99)	2.06(0.88,4.83)	
Non-Hispanic black	Reference	1.08(0.72, 1.61)	1.68(1.08, 2.61)	8.85(2.69,29.14)	3.15(1.71, 5.78)	3.18(2.22, 4.55)	
Non-Hispanic white	Reference	2.23(1.54,3.23)	2.61(1.90,3.58)	2.89(1.26,6.62)	4.38(2.97,6.47)	5.16(3.46,7.68)	
Other Hispanic		1.33(0.59,2.97)	1.36(0.52,3.59)	0.00(0.00,0.00)	1.08(0.37,3.15)	2.44(1.12,5.33)	
Other Race		1.20(0.57, 2.51)	1.01(0.43, 2.39)	2.56(0.56,11.81)	2.16(0.90, 5.16)	3.30(1.56, 6.99)	
**Smoking status**							0.09
Never	Reference	2.20(1.43,3.39)	2.56(1.69,3.88)	2.00(0.50,8.00)	5.20(3.12,8.65)	4.54(2.90,7.12)	
Former	Reference	2.61(1.64, 4.15)	2.90(1.95, 4.30)	3.14(1.18, 8.35)	4.12(2.45, 6.93)	6.63(4.16,10.54)	
Now	Reference	1.24(0.82,1.89)	1.71(1.07,2.74)	3.85(1.66,8.92)	2.48(1.55,3.96)	3.40(2.20,5.23)	
**Drinking status**							0.34
Never	Reference	2.46(1.12,5.38)	2.18(1.00,4.74)	2.19(0.59,8.08)	3.97(2.05,7.67)	3.94(1.88,8.26)	
Former	Reference	1.08(0.73,1.59)	1.32(0.90,1.93)	3.52(1.32,9.41)	2.04(1.29,3.20)	2.72(1.90,3.91)	
Now	Reference	1.94(1.37,2.76)	2.39(1.78,3.21)	2.87(1.24,6.66)	4.02(2.80,5.79)	4.82(3.43,6.79)	
**Physical activity**							0.58
Inactive	Reference	1.89(1.39,2.58)	2.46(1.81,3.35)	4.70(2.45,9.04)	3.91(2.57,5.94)	4.54(3.33,6.19)	
Active	Reference	1.77(1.17,2.69)	1.89(1.31,2.72)	1.76(0.64,4.85)	3.40(2.17,5.32)	4.23(2.77,6.48)	

MHN, metabolically healthy normal weight; MHOW, metabolically healthy overweight; MHO, metabolically healthy obesity; MUN, metabolically unhealthy normal weight; MUOW, metabolically unhealthy overweight; MUO, metabolically unhealthy obesity; CVD, cardiovascular disease.

Model was adjusted for age, sex, ethnicity, educational level, marital status, smoking status, drinking status, and physical activity.

## Discussion

In this population-based study, individuals with MUO had the highest prevalence of composite CVD. The MUO is the strongest independent risk factor of composite CVD and five CVD events, regardless of whether the diagnosis of obese status on metabolic phenotype was defined as body fat or BMI categories. Compared with MHN, MHO is also an independent risk factor for CVD. A non-linear dose-response relationship between BFP and CVD was observed both in metabolically healthy and unhealthy participants. The associations were robust in different subgroups of age, sex, ethnicity, smoking, drinking status, and physical activity, with only significant interaction of metabolic phenotype with sex. These findings suggested that metabolic phenotypes can independently contribute to CVD.

There are numerous studies being conducted on the risk of CVD among individuals with different metabolic phenotypes. However, these studies have produced conflicting results. One possible reason for this heterogeneity is the use of different definitions for metabolic status and obese status. Several recent studies report that MHO is not a completely benign condition, with regards to the difference in associations between MHN, MHO and CVD incidence [24, 25]. Two meta-analyses of prospective cohort studies that included more than 4.4 million participants discovered that the risk of CVD was higher in metabolically healthy groups with overweight (relative risk: 1.34) and obesity (relative risk: 1.50–1.58) as compared to those who were metabolically healthy and had normal weight [13, 24]. These findings were consistent with our research, however, several studies have reported otherwise [17, 26, 27].

In a cohort study of European adults, Appleton et al. discovered that individuals with MHO did not experience an increased risk of cardiovascular disease (CVD) [26]. Similarly, Hamer M et al. found no heightened CVD risk among 22203 MHO participants in Scotland and England [27]. However, the contradictory results may be attributed to the disparate diagnostic criteria of metabolic health, as well as the relatively small sample size and diverse ethnicities. It’s important to note that previous studies often classified obesity based on BMI. However, BMI has been criticized for its inability to distinguish between lean and fat mass. To overcome this limitation, DXA has become a standard assessment method for body composition evaluation as it provides high precision and simplicity, making it widely used in clinics. In this study, obesity was defined based on BFP assessed by DXA. The results showed that BFP is associated with an increased risk of CVD, which is consistent with the findings of Ortega et al. who assessed BFP with skinfolds [28]. This study provides new information about how metabolic obesity phenotypes impact the risk of CVD. By examining BMI categories, the study found that both MHO and MUO are independently associated with a higher risk of CVD. This emphasizes the negative impact of metabolic unhealthiness and excess body fat on CVD incidence. These findings have important implications for public health policy regarding CVD prevention. The study used a large sample size of over 40000 US participants, making the results consistent with previous studies that show individuals with both MHO and MUO have higher risks of CVD than those who are metabolically healthy and have a normal weight.

Our study found a relatively J-shaped association between BFP and CVD risk among general participants, which was similar with a previous study [29, 30]. A dose-response meta-analysis of 35 prospective cohort studies showed that there was a J shaped association between BFP and all-cause mortality, with the lowest risk at BFP of 25% [29]. Another meta-analysis of 1 million adults demonstrated a J-shaped association between adiposity, defined by BMI, waist circumference, or waist-hip ratio, and risk of incident heart failure [30]. A similar J-shaped association has also been found among participants with metabolically unhealthy. However, we found that a relatively U-shaped association between BFP and CVD among participants with metabolically healthy with threshold range of 22.4%-40.8%. Our results extended the relationship between BFP and CVD.

This study has some limitations that should be taken into consideration. Firstly, due to its cross-sectional design, it was not possible to establish a causal link. Secondly, even though all the critical factors were adjusted, there could still be confounders that were not accounted for in the analysis, which could produce biased associations. Thirdly, all the participants in the study were individuals from the US, which may limit the generalizability of the findings. Finally, the metabolic phenotype was analyzed as a transient status, and changes in this status may also affect the prevalence of CVD. Further research could use the MHO model to understand how obesity, adipose tissue expansion, cellular composition, and dysfunction contribute to obesity-associated CVD. Randomized control trials are urgently needed to determine whether individuals with MHO are at a lower risk of developing CVD compared to those with MUO.

## Conclusions

In this study, obesity is a significant risk factor for developing CVD in both healthy and unhealthy individuals. This risk is not only limited to composite CVD but also includes stroke, CHD, angina, and heart attack. Whether using BFP or BMI to define obesity, the associations between metabolic obesity status and CVD risk are similar, which imply that BFP is a precise indicator to predict obesity-related CVD outcomes. Therefore, it is crucial to improve both metabolic health and obese status to reduce the risk of developing CVD.

## Supporting information

S1 Table BFP criteria used to determine participants’ obesity category. (DOCX)

S2 Table Associations between metabolic obesity phenotypes (defined by BMI) and CVD risk. (DOCX)

S3 Table Subgroup analyses of association between metabolic obesity phenotypes (defined by BMI) and CVD risk. (DOCX)

## References

[1] Global burden of 369 diseases and injuries in 204 countries and territories, 1990–2019: a systematic analysis for the Global Burden of Disease Study 2019. *Lancet*. 2020;396:1204–1222. doi: 10.1016/S0140-6736(20)30925-9 33069326 PMC7567026

[2] Roth GA, Mensah GA, Johnson CO, Addolorato G, Ammirati E, Baddour LM, et al. Global burden of cardiovascular diseases and risk factors, 1990–2019: update from the GBD 2019 study. *J Am Coll Cardiol*.2020;76:2982–3021. doi: 10.1016/j.jacc.2020.11.010 33309175 PMC7755038

[3] World Health Organization. *Obesity and overweight*. https://www.who.int/news-room/fact-sheets/detail/obesity-and-overweight (Accessed August 17, 2023).

[4] Lavie CJ, Laddu D, Arena R, Ortega FB, Alpert MA, Kushner RF. Healthy weight and obesity prevention: *JACC* health promotion series. *J Am Coll Cardiol*. 2018;72:1506–1531. 30236314 10.1016/j.jacc.2018.08.1037

[5] Berrington de Gonzalez A, Hartge P, Cerhan JR, Hannan L, MacInnis RJ, Moore SC, et al. Body-mass index and mortality among 1.46 million white adults. *N Engl J Med*.2010;363:2211–2219. doi: 10.1056/NEJMoa1000367 21121834 PMC3066051

[6] GBD 2015 Obesity Collaborators, Afshin A, Forouzanfar MH, et al. Health effects of overweight and obesity in 195 countries over 25 years. *N Engl J Med*. 2017;377:13–27. doi: 10.1056/NEJMoa1614362 28604169 PMC5477817

[7] Jarolimova J, Tagoni J, Stern TA. Obesity: its epidemiology, comorbidities, and management. *Prim Care Companion CNS Disord*. 2013;15 PCC.12f01475. doi: 10.4088/PCC.12f01475 24511434 PMC3907314

[8] Meigs JB, Wilson PW, Fox CS, Vasan RS, Nathan DM, Sullivan LM, et al. Body mass index, metabolic syndrome, and risk of type 2 diabetes or cardiovascular disease. *J Clin Endocrinol Metab*.2006;91:2906–2912. doi: 10.1210/jc.2006-0594 16735483

[9] Kissebah AH, Vydelingum N, Murray R, Evans DJ, Hartz AJ, Kalkhoff RK, et al. Relation of body fat distribution to metabolic complications of obesity. *J Clin Endocrinol Metab*.1982;54:254–260. doi: 10.1210/jcem-54-2-254 7033275

[10] Mirzaei B, Abdi H, Serahati S, Barzin M, Niroomand M, Azizi F, et al. Cardiovascular risk in different obesity phenotypes over a decade follow-up: Tehran lipid and glucose study. *Atherosclerosis*.2017;258:65–71. doi: 10.1016/j.atherosclerosis.2017.02.002 28213199

[11] Liu JT, Yao HY, Yu SC, Liu JJ, Zhu GJ, Han SM, et al. Joint association of metabolic health and obesity with ten-year risk of cardiovascular disease among Chinese adults. *BioMed Environ Sci*.2022;35:13–21. doi: 10.3967/bes2022.003 35078558

[12] Zhang X, Zhu J, Kim JH, Sumerlin TS, Feng Q, Yu J. Metabolic health and adiposity transitions and risks of type 2 diabetes and cardiovascular diseases: a systematic review and meta-analysis. *Diabetol Metab Syndr*. 2023;15:60. doi: 10.1186/s13098-023-01025-w 36973730 PMC10045173

[13] Opio J, Croker E, Odongo GS, Attia J, Wynne K, McEvoy M, et al. Metabolically healthy overweight/obesity are associated with increased risk of cardiovascular disease in adults, even in the absence of metabolic risk factors: a systematic review and meta-analysis of prospective cohort studies. *Obes Rev*.2020;21:e13127. doi: 10.1111/obr.13127 32869512

[14] Kramer CK, Zinman B, Retnakaran R. Are metabolically healthy overweight and obesity benign conditions?: a systematic review and meta-analysis. *Ann Intern Med*.2013;159:758–769. doi: 10.7326/0003-4819-159-11-201312030-00008 24297192

[15] Fan J, Song Y, Chen Y, Hui R, Zhang W. Combined effect of obesity and cardio-metabolic abnormality on the risk of cardiovascular disease: a meta-analysis of prospective cohort studies. *Int J Cardiol*.2013;168:4761–4768. doi: 10.1016/j.ijcard.2013.07.230 23972953

[16] Eckel N, Meidtner K, Kalle-Uhlmann T, Stefan N, Schulze MB. Metabolically healthy obesity and cardiovascular events: a systematic review and meta-analysis. *Eur J Prev Cardiol*.2016;23:956–966. doi: 10.1177/2047487315623884 26701871

[17] Zembic A, Eckel N, Stefan N, Baudry J, Schulze MB. An empirically derived definition of metabolically healthy obesity based on risk of cardiovascular and total mortality. *JAMA Netw Open*.2021;4:e218505. doi: 10.1001/jamanetworkopen.2021.8505 33961036 PMC8105750

[18] Cornier MA, Després JP, Davis N, et al. Assessing adiposity: A scientific statement from the American Heart Association. *Circulation*. 2011;124:1996–2019. doi: 10.1161/CIR.0b013e318233bc6a 21947291

[19] Ntougou Assoumou HG, Pichot V, Barthelemy JC, Dauphinot V, Celle S, Collet P, Gaspoz JM. Roche Frederic. Obesity-related autonomic nervous system disorders are best associated with body fat mass index, a new indicator. *Int J Cardio**l*. 2011;153:111–113. doi: 10.1016/j.ijcard.2011.09.031 21963211

[20] Expert Panel on Detection Evaluation and Treatment of High Blood Cholesterol in Adults. Executive summary of the third report of the national cholesterol education program (NCEP) expert panel on detection, evaluation, and treatment of high blood cholesterol in adults (adult treatment panel III). *JAMA*. 2001;285:2486–2497 doi: 10.1001/jama.285.19.2486 11368702

[21] Bray GA. Contemporary diagnosis and management of obesity and the metabolic syndrome. 3rd ed. Newtown, PA: Handbooks in Health Care Co (2003).

[22] World Health Organization (WHO). Obesity: preventing and managing the global epidemic In: Report on a WHO consultation on obesity. Geneva: World Health Organization (1997). 11234459

[23] https://r-survey.r-forge.r-project.org/survey.

[24] Zheng R, Zhou D, Zhu Y. The long-term prognosis of cardiovascular disease and all-cause mortality for metabolically healthy obesity: a systematic review and meta-analysis. *J Epidemiol Community Health*. 2016;70:1024–1031. doi: 10.1136/jech-2015-206948 27126492

[25] Eckel N, Li Y, Kuxhaus O, Stefan N, Hu FB, Schulze MB. Transition from metabolic healthy to unhealthy phenotypes and association with cardiovascular disease risk across BMI categories in 90 257 women (the Nurses’ Health Study): 30-year follow-up from a prospective cohort study. *Lancet Diabetes Endocrinol*. 2018;6:714–724. 29859908 10.1016/S2213-8587(18)30137-2

[26] Appleton SL, Seaborn CJ, Visvanathan R, et al. Diabetes and cardiovascular disease outcomes in the metabolically healthy obese phenotype: a cohort study. *Diabetes Care*. 2013;36:2388–2394. doi: 10.2337/dc12-1971 23491523 PMC3714523

[27] Hamer M, Stamatakis E. Metabolically healthy obesity and risk of all-cause and cardiovascular disease mortality. *J Clin Endocrinol Metab*.2012;97:2482–2488. doi: 10.1210/jc.2011-3475 22508708 PMC3387408

[28] Ortega FB, Lee DC, Katzmarzyk PT, et al. The intriguing metabolically healthy but obese phenotype: cardiovascular prognosis and role of fitness. *Eur Heart J*. 2013;34:389–397. doi: 10.1093/eurheartj/ehs174 22947612 PMC3561613

[29] Jayedi A, Khan TA, Aune D, et al. Body fat and risk of all-cause mortality: a systematic review and dose-response meta-analysis of prospective cohort studies. *Int J Obes (Lond)*. 2022 Sep;46(9):1573–1581. doi: 10.1038/s41366-022-01165-5 35717418

[30] Oguntade AS, Islam N, Malouf R, et al. Body Composition and Risk of Incident Heart Failure in 1 Million Adults: A Systematic Review and Dose-Response Meta-Analysis of Prospective Cohort Studies. *J Am Heart Assoc*. 2023 Jul 4;12(13):e029062. doi: 10.1161/JAHA.122.029062 37345755 PMC10356078

