# Supplementary material for: Comparison of cardiovascular disease risk association with metabolic unhealthy obesity identified by body fat percentage and body mass index: Results from the 1999–2020 National Health and Nutrition Examination Survey
Source: PLoS One. 2024 Aug 14;19(8):e0305592. doi: 10.1371/journal.pone.0305592 (PMC11324142; doi:10.1371/journal.pone.0305592)
Supplement: S1 Table — (DOCX) [file pone.0305592.s001.docx]

**Table S1. BFP criteria used to determine participants’ obesity category**

| **Category** | **Male** | |  | **Female** | |
| --- | --- | --- | --- | --- | --- |
|  | **20-39 years** | **>40 years** |  | **20-39 years** | **>40 years** |
| Underweight | < 8.0 | < 11.0 |  | < 21.0 | < 23.0 |
| Normal weight | 8.0-20.9 | 11.0-22.9 |  | 21.0-32.9 | 23.0-34.9 |
| Overweight | 21.0-25.9 | 23.0-28.9 |  | 33.0-38.9 | 35.0-40.9 |
| Obesity | > 26.0 | > 29.0 |  | > 39.0 | > 41.0 |
