# Supplementary material for: Comparison of cardiovascular disease risk association with metabolic unhealthy obesity identified by body fat percentage and body mass index: Results from the 1999–2020 National Health and Nutrition Examination Survey
Source: PLoS One. 2024 Aug 14;19(8):e0305592. doi: 10.1371/journal.pone.0305592 (PMC11324142; doi:10.1371/journal.pone.0305592)
Supplement: S2 Table — (DOCX) [file pone.0305592.s002.docx]

**Table S2. Associations between metabolic obesity phenotypes (defined by BMI) and CVD risk**

| **Variables** | **MHN** | **MHOW** | **MHO** | **MUN** | **MUOW** | **MUO** |
| --- | --- | --- | --- | --- | --- | --- |
| **CVD** |  |  |  |  |  |  |
| Model 1 | Reference | 1.41(1.16,1.72) | 1.26(1.07,1.49) | 6.45(4.59,9.07) | 4.37(3.49,5.47) | 4.00(3.20,5.00) |
| Model 2 | Reference | 1.21(0.98,1.51) | 1.36(1.13,1.64) | 2.82(1.93,4.13) | 2.15(1.70,2.72) | 2.99(2.39,3.75) |
| Model 3 | Reference | 1.29(1.02,1.62) | 1.45(1.19,1.75) | 2.65(1.74,4.05) | 2.10(1.65,2.67) | 2.98(2.38,3.74) |
| **Stroke** |  |  |  |  |  |  |
| Model 1 | Reference | 1.31(0.96,1.79) | 1.27(0.97,1.66) | 4.25(2.96,6.09) | 3.25(2.31,4.57) | 3.23(2.48,4.20) |
| Model 2 | Reference | 1.20(0.88,1.64) | 1.35(1.04,1.74) | 1.77(1.23,2.53) | 1.67(1.20,2.34) | 2.35(1.80,3.07) |
| Model 3 | Reference | 1.28(0.93,1.78) | 1.41(1.09,1.82) | 1.57(1.08,2.28) | 1.62(1.14,2.30) | 2.34(1.79,3.06) |
| **CHD** |  |  |  |  |  |  |
| Model 1 | Reference | 1.63(1.18, 2.24) | 0.95(0.66, 1.36) | 9.07(5.22,15.75) | 5.13(3.94, 6.68) | 5.05(3.52, 7.23) |
| Model 2 | Reference | 1.33(0.94,1.87) | 1.04(0.70,1.55) | 4.13(2.27,7.53) | 2.35(1.80,3.05) | 3.72(2.56,5.41) |
| Model 3 | Reference | 1.36(0.96,1.92) | 1.08(0.72,1.60) | 4.17(2.18,8.00) | 2.30(1.77,2.99) | 3.66(2.57,5.20) |
| **CHF** |  |  |  |  |  |  |
| Model 1 | Reference | 1.27(0.85, 1.91) | 1.79(1.38, 2.34) | 6.72(4.41,10.24) | 4.83(3.73, 6.24) | 6.50(4.97, 8.51) |
| Model 2 | Reference | 1.07(0.71,1.62) | 1.96(1.48,2.61) | 2.83(1.78,4.50) | 2.26(1.72,2.98) | 4.71(3.62,6.14) |
| Model 3 | Reference | 1.13(0.75,1.70) | 2.03(1.52,2.72) | 2.48(1.59,3.88) | 2.17(1.63,2.88) | 4.49(3.46,5.82) |
| **Heart attack** |  |  |  |  |  |  |
| Model 1 | Reference | 1.51(1.15,1.99) | 1.18(0.89,1.55) | 5.59(4.01,7.80) | 4.62(3.57,5.96) | 4.42(3.42,5.71) |
| Model 2 | Reference | 1.23(0.92,1.63) | 1.24(0.92,1.66) | 2.60(1.79,3.77) | 2.22(1.69,2.92) | 3.17(2.46,4.07) |
| Model 3 | Reference | 1.34(1.01,1.80) | 1.38(1.03,1.85) | 2.46(1.72,3.51) | 2.18(1.64,2.89) | 3.22(2.56,4.05) |
| **Angina** |  |  |  |  |  |  |
| Model 1 | Reference | 1.45(1.00, 2.10) | 1.27(0.90, 1.79) | 7.21(3.10,16.75) | 4.20(2.91, 6.06) | 4.38(3.14, 6.12) |
| Model 2 | Reference | 1.22(0.85,1.75) | 1.33(0.94,1.90) | 3.31(1.35,8.12) | 2.09(1.46,3.00) | 3.14(2.29,4.31) |
| Model 3 | Reference | 1.29(0.89,1.86) | 1.46(1.02,2.10) | 3.07(1.17,8.05) | 2.01(1.39,2.91) | 3.11(2.25,4.30) |

MHN, metabolically healthy normal weight; MHOW, metabolically healthy overweight; MHO, metabolically healthy obesity; MUN, metabolically unhealthy normal weight; MUOW, metabolically unhealthy overweight; MUO, metabolically unhealthy obesity; CVD, cardiovascular disease; CHD, coronary heart disease; CHF, congestive heart failure.

Model 1, unadjusted; Model 2, adjusted for age, and sex; Model 3, adjusted for age, sex, ethnicity, educational level, marital status, smoking status, drinking status, and physical activity.
