# Supplementary material for: Comparison of cardiovascular disease risk association with metabolic unhealthy obesity identified by body fat percentage and body mass index: Results from the 1999–2020 National Health and Nutrition Examination Survey
Source: PLoS One. 2024 Aug 14;19(8):e0305592. doi: 10.1371/journal.pone.0305592 (PMC11324142; doi:10.1371/journal.pone.0305592)
Supplement: S3 Table — (DOCX) [file pone.0305592.s003.docx]

**Table S3. Subgroup analyses of association between metabolic obesity phenotypes (defined by BMI) and CVD risk**

| **Variables** | **MHN** | **MHOW** | **MHO** | **MUN** | **MUOW** | **MUO** | ***P* for**  **interaction** |
| --- | --- | --- | --- | --- | --- | --- | --- |
| **Age** |  |  |  |  |  |  | 0.09 |
| ≥40 years | Reference | 1.14(0.92,1.42) | 1.16(0.97,1.38) | 3.47(2.32,5.20) | 2.39(1.87,3.06) | 2.45(1.93,3.11) |  |
| <40 years | Reference | 1.26(0.74, 2.12) | 1.36(0.78, 2.38) | 3.40(0.83,13.84) | 0.95(0.27, 3.34) | 4.95(2.55, 9.59) |  |
| **Sex** |  |  |  |  |  |  | 0.11 |
| Female | Reference | 1.16(0.79,1.70) | 1.47(1.14,1.91) | 3.31(2.37,4.62) | 2.60(1.80,3.78) | 3.02(2.06,4.44) |  |
| Male | Reference | 1.08(0.85,1.38) | 0.91(0.67,1.25) | 4.07(1.84,9.03) | 2.15(1.68,2.75) | 2.13(1.69,2.68) |  |
| **Ethnicity** |  |  |  |  |  |  | 0.17 |
| Mexican American | Reference | 0.64(0.36,1.13) | 0.70(0.38,1.29) | 1.59(0.84,3.04) | 1.89(1.22,2.93) | 1.37(0.76,2.48) |  |
| Non-Hispanic black | Reference | 0.93(0.67,1.30) | 1.25(0.88,1.77) | 4.99(2.69,9.26) | 2.33(1.67,3.24) | 2.46(1.90,3.18) |  |
| Non-Hispanic white | Reference | 1.33(1.03,1.71) | 1.27(0.99,1.62) | 3.75(2.27,6.20) | 2.54(1.89,3.42) | 2.83(2.15,3.72) |  |
| Other Hispanic |  | 0.76(0.42,1.37) | 0.65(0.27,1.57) | 0.91(0.34,2.43) | 1.38(0.73,2.61) | 1.31(0.73,2.34) |  |
| Other Race |  | 0.45(0.25,0.81) | 0.74(0.37,1.50) | 3.40(1.70,6.81) | 2.11(1.06,4.19) | 1.81(1.01,3.24) |  |
| **Smoking status** |  |  |  |  |  |  | 0.52 |
| Never | Reference | 1.27(0.92,1.75) | 1.13(0.84,1.52) | 3.84(1.86,7.89) | 2.55(1.94,3.36) | 2.27(1.73,2.99) |  |
| Former | Reference | 1.08(0.78,1.49) | 1.13(0.84,1.52) | 3.04(1.97,4.70) | 2.38(1.62,3.51) | 2.78(1.90,4.08) |  |
| Current | Reference | 1.06(0.74,1.53) | 1.42(0.91,2.22) | 3.64(2.28,5.82) | 2.27(1.44,3.57) | 2.91(2.07,4.08) |  |
| **Drinking status** |  |  |  |  |  |  | 0.86 |
| Never | Reference | 1.27(0.82,1.99) | 1.35(0.81,2.27) | 3.40(2.07,5.56) | 2.35(1.68,3.29) | 2.12(1.30,3.46) |  |
| Former | Reference | 0.92(0.70,1.19) | 0.81(0.61,1.08) | 2.85(1.80,4.51) | 1.97(1.47,2.62) | 2.01(1.57,2.57) |  |
| Current | Reference | 1.21(0.92,1.58) | 1.25(0.98,1.58) | 3.77(2.08,6.83) | 2.54(1.89,3.43) | 2.79(2.19,3.55) |  |
| **Physical activity** |  |  |  |  |  |  | 0.62 |
| Inactive | Reference | 1.18(0.93,1.51) | 1.14(0.92,1.41) | 4.04(2.45,6.66) | 2.41(1.82,3.18) | 2.42(1.96,3.00) |  |
| Active | Reference | 1.14(0.84,1.55) | 1.25(0.94,1.68) | 2.78(1.81,4.27) | 2.50(1.79,3.50) | 2.84(2.05,3.95) |  |

MHN, metabolically healthy normal weight; MHOW, metabolically healthy overweight; MHO, metabolically healthy obesity; MUN, metabolically unhealthy normal weight; MUOW, metabolically unhealthy overweight; MUO, metabolically unhealthy obesity; CVD, cardiovascular disease.

Model was adjusted for age, sex, ethnicity, educational level, marital status, smoking status, drinking status, and physical activity.
